# Metabolic Syndrome Is a Strong Risk Factor for Minor Ischemic Stroke and Subsequent Vascular Events

**DOI:** 10.1371/journal.pone.0156243

**Published:** 2016-08-18

**Authors:** Guang-Sheng Wang, Dao-Ming Tong, Xiao-Dong Chen, Tong-Hui Yang, Ye-Ting Zhou, Xiao-Bo Ma

**Affiliations:** 1 Department of Neurology, Affiliated Shu Yang People′s Hospital, XuZhou Medical University, XuZhou, China; 2 Department of Clinical Research, Affiliated Shu Yang People's Hospital, XuZhou Medical University, XuZhou, China; 3 Department of science and education, Affiliated Shu Yang People′s Hospital, XuZhou Medical University, XuZhou, China; Universita degli Studi di Perugia, ITALY

## Abstract

**Background:**

Minor ischemic stroke (MIS) represents a major global public health problem worldwide due to high incidence. The aim of this study was to investigate whether metabolic syndrome (MetS) is a strong risk for MIS and subsequent vascular events (SVE).

**Methods:**

A retrospective cohort study was performed examining symptomatic MIS in a Chinese neurologic outpatient population aged over 25 years without history of stroke. MetS was defined using the International Diabetes Federation criteria. MIS was diagnosed by magnetic resonance imaging-diffusion weighted images or fluid-attenuated inversion recovery.

**Results:**

Of 1361 outpatients, a total of 753 (55.3%) patients were diagnosed with MIS; of them, 80% had a score of 0 using the MIS had a 0 score on the National Institutes of Health Stroke Scale. Among these, 303 (40.2%) individuals with MIS were diagnosed with MetS. Diagnosed of MIS with MetS significantly correlated with abdominal obesity (30.7% v.s 18.0%), hypertension (91.1% v.s 81.6%), increased blood glucose (6.9±2.4 v.s 5.0±0.4), dyslipidemia (78.2% v.s 48.2%), and SVE (50.5% v.s 11.3%) when compared with the controls group. On adjusted analysis, the risk of SVE was also significantly associated with three additional MetS criterion (RR,9.0; 95% CI, 5.677–14.46). Using Cox proportional analysis, risk of SVE in patient with MIS was significantly associated with MetS (RR, 3.3; 95% CI, 1.799–6.210), older age (RR, 1.0; 95% CI, 1.001–1.048), and high blood glucose (RR,1.1; 95%CI, 1.007–1.187).

**Conclusions:**

The MetS is a strong risk factor for MIS, and patients presenting with MIS and MetS are at a high risk of SVE. Further studies are required to determine the improvement of Mets prevention in the reduction of MIS and SVE.

## Introduction

Minor ischemic stroke (MIS) has become an important public health problem, particularly as several epidemiological studies have reported an incidence of up to 55%-74% in metropolitan regions [1], [2]. Recently, studies have demonstrated that many well-known vascular risk factors such as hypertension, smoking, diabetes, cholesterolemia, atrial fibrillation, heart disease, and being overweight or obese remain significantly associated with ischemic stroke in both the general population and those at high risk of stroke [3], [4]. In addition, some new risk factors, including dyslipidemia, increased blood levels of C-reactive protein, carotid intima-media thickness, metabolic syndrome (MetS), older age, and gender have been identified [4], [5]. These risk factors increase the risk of cardiovascular diseases and ischemic strokes [6]–[8]. In particular, abdominal obesity, dyslipidemia, hyperglycemia, and symptomatic hypertension are together regarded as the four important risk factors for ischemic stroke and are referred to as MetS [5], [8–10]. Previous hospital based studies have confirmed that MetS is not only a risk factor for intracranial atherosclerotic stroke [11], [12], but it is also more likely to cause acute severe ischemic stroke [13–15]. Moreover, the important role of inflammation and arterial stiffness has been demonstrated in acute ischemic stroke patients with MetS [16], [17]. However, few studies have assessed the role of MetS as a strong risk factor for MIS, and there is limited data showing that these patients are more likely to present with subsequent vascular events (SVE). Thus, this retrospective cohort study analyzed data from neurologic outpatients without history of stroke. The overall aim was to assess whether MetS was associated with increased risk of MIS and SVE.

## Methods

### Ethics Statement

The study was approved by the Ethical Committee on Clinical Research of the Shuyang People' hospital, China. The study was in full compliance with the Helsinki declaration, and solely required de-identification of all personal information related to the already collected clinical data without requirement of informed consent.

### Study population

A population-based retrospective cohort study was performed recruiting consecutive new patients for assessment between January 2011 and February 2013. All were registered neurologic outpatients at a tertiary teaching hospital in northern Jiangsu, China. Symptomatic outpatients(n = 1361), clinically only having had their initial visit and having undergone a magnetic resonance imaging (MRI) study of the brain, were enrolled. To be eligible, subjects were required to be >25 years old, and live in one of 38 villages or towns, or one urban center in Shu Yang of northern China. Included patients were also required to have a follow-up assessment at 6 months.

### Assessment of minor ischemic stroke

Based on radiographic imaging, ischemic infarct can be divided into either a lacunar stroke or non-lacunar stroke. MIS is usually indicated by the lacunar stroke subtype, due to small vessel disease (SVD), although this is not always the case. However, the trial of ORG 10172 in acute stroke treatment denoted five ischemic stroke subtypes, and these may also contribute to MIS. These include arterothrombotic MIS, cardioembolic MIS, and non-SVD MIS due to another etiology. Therefore, for the purposes of this study, the inclusion criteria of MIS were as follows: (1) first visit indicating minor brain symptoms, with or without minor positive signs of stroke, and measured as ≤3 on the National Institutes of Health Stroke Scale (NIHSS) [18]; (2) an MRI displaying visible MIS as a small infarcts within 24 hours of initial presentation [19], or lacunar lesions [20]or increased brain signal using diffusion weighted imaging (DWI) or fluid-attenuated inversion recovery (FLAIR), with a location in the subcortical white matter, the basal ganglia, or the brain stem. Exclusion criteria were as follows: (1) age <25 years; (2) previous history of stroke diagnosed; or (3) an NIHSS score of >3 on first visit.

### Definition of the MetS

Subjects were diagnosed with MetS if they met at least three of the following revised American Heart Association/National Heart, Lung, and Blood Institute Scientific Statement criteria [12]: (1)an elevated waist circumference of >102 cm for men and >88 cm for women; (2) elevated triglycerides >150 mg/dL(1.7mmol/l); (3) reduced high-density lipoprotein cholesterol of <40 mg/dL(1.03mmol/l) for men and <50 mg/dL(1.3mmol/l) for women; (4) elevated blood pressure ≥130 mm Hg systolic blood pressure or ≥80 mm Hg diastolic blood pressure; (5) elevated fasting blood glucose ≥100 mg/dL(5.6mmol/l).

For Asian populations, the International Diabetes Foundation thresholds for abdominal obesity were used: waist circumferences ≥90 cm in men and ≥80 cm in women [12].

### Determination of subsequent vascular event (SVE)

SVE was defined as a transient ischemic attack (TIA), the presence of transient nonfocal cerebrovascular symptoms, deterioration of cerebrovascular symptoms, or recurrent infarction, in addition to a Rankin scale score≥ 2 during a follow-up visit.

### Data Collection

Throughout the study, trained researchers utilized instruments to collect data. Anthropometric parameters and blood pressure were measured by standard methods. Fasting plasma glucose and lipid profiles were measured in a central laboratory. The following vascular risk factors were obtained from registered outpatients. Current smokers were defined as those who had smoked at least one cigarette per day during the 12 months prior to assessment. Excessive alcohol intake was defined as consumption of ≥30 g of alcohol per day. Baseline BMI was categorized as normal (BMI < 25 kg/m2), overweight (25–30 kg/m2), or obese (greater than 30 kg/m2). Waist circumference was measured if the patient had an overweight or obese. Hypertension was defined as having a clinical history of hypertension, or 2 or more previously documented systolic blood pressures greater than 160 mmHg or diastolic blood pressures greater than 90 mmHg. Diabetes mellitus was deemed present if the patient gave a history of diabetes that was confirmed by medical records, or had a blood glucose concentration greater than 11 mmol/l. Dyslipidemia was deemed present if total venous plasma cholesterol levels were greater than 6.0 mmol/l, the low-density lipoprotein fraction was greater than 3.0 mmol/l, triglyceride levels were greater than 1.7 mmol/l, and high density lipoprotein cholesterol less than 1.03mmol/l in males or less than 1.3 mmol/l in females. Coronary heart disease was defined as a history of an acute myocardial infarction or angina pectoris. Atrial fibrillation, as indicated by an electrocardiogram during the initial visit, or by medical records.

All patients underwent a brain MRI within 24 hours of the first visit. The MRI was performed with 1.5-T equipment (Siemens). All MRIs were reviewed by a neuroradiologist and a neurologist who were blinded to the study. The examiners specifically assessed the presence of absence of hyperintense lesions using FLAIR or DWI. The maximum diameters (in mm), number, and location of lesions were recorded in detail for each patient.

The NIHSS criteria were used to assess the symptom severity of each outpatient at the time of initial visit. All patients with MIS or TIA were given aspirin therapy (100 mg daily). Hypertension was treated with clinically appropriate agents. Patients diagnosed diabetes mellitus were provided with specialist treatment as required. In order to further investigate MIS patients with or without SVE during the 180-day follow-up assessment, information was gathered by a neurological specialist by telephone. For patients who died during follow-up, information was obtained from relatives and/or hospital records.

### Statistical analyses

Statistical analyses were carried out using SPSS version 17.0 (SPSS Inc., Chicago, IL, USA). Discrete data are presented as numbers and percentages, means and standard deviations, medians and interquartile ranges, or proportions with 95% confidence intervals. Accordingly, chi-square tests were summarized as numbers with percentages. Independent *t* test and Mann-Whitney *U* test were used to compare differences in continuous variables between the two groups. Chi-squared tests and Pearson's correlation coefficients were used to explore the relationships between baseline variables. Age-and multivariate-adjusted risk ratios (RR) and 95% confidence intervals (CIs) were estimated with the use of the logistic-regression model or Cox proportional hazards model to examine MetS baseline status and determine whether the variables played a role in the risk of incident MIS and its SVE. For all statistical analyses, a two-sided *P*<0.05 was considered statistically significant.

## Results

A total of 1361 subjects enrolled in this study. Of these, 608 patients were excluded. 268 patients with a final diagnosis of TIA, 139 patients with primary migraine without infarction, 137 patients with peripheral vertigo, 51 patients with other brain episodes, and 13 patients with infarction and an initial NIHSS score>3. Eventually, 753 outpatients with a mean age of 60±11.6 years (range: 25–84 years) were included in the investigation.

**Fig 1:**
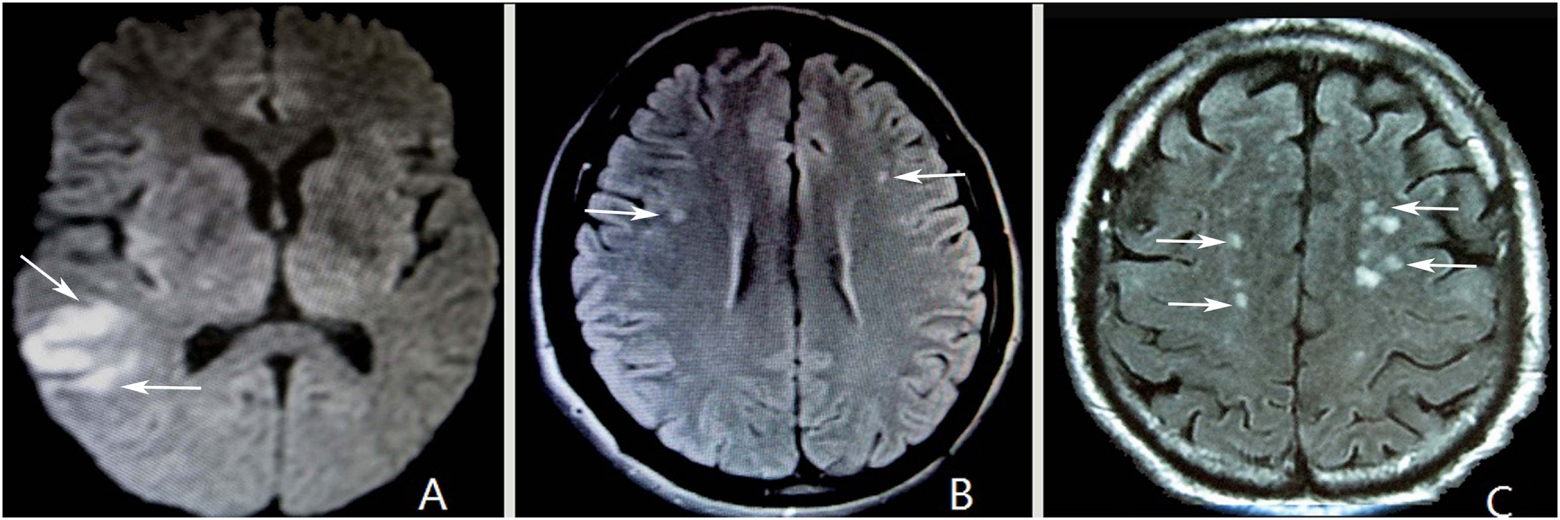
Nonfocal symptoms in MIS patients with or without MetS on brain MRI. A 65-year-old female with headache and dizziness for 3d (NIHSS = 0 score), several typical small infarcts in the right lateral temporal and parietal area is visible on the DWI (A, arrows), and she were diagnosed with MIS with MetS. A 52-year-old male with dizziness for 2 d (NIHSS = 0) and with MetS, on the FLAIR imaging shows two recent small infarcts near the centrum ovale areas (B, arrows). A 76-year-old male with recurrent headache for 10 d (NIHSS = 0) without MetS, MRI-FLAIR shows typical recent multiple small infarcts in bilateral anterior circulation areas (C, arrows).

Baseline characteristics of the patients with MIS are shown in Table 1. 753 patients with MIS were diagnosed using MRI-FLAIR or DWI within 24 hours of the initial visit (the execution rate of DWI only up to 53%). Of them, 80% of patients with MIS had a score of 0 using NIHSS criteria, while only 20% had scores of 1–3. The overall prevalence of MIS at baseline was 55.3%. The main type of symptom in patients with MIS were non-focal neurologic symptoms(90%) and a large proportion of these patients had multiple small infarcts(91.6%) on MRI (Fig 1). The most common affected vascular territory was in the anterior circulation artery (ACA) regions (55.5%), followed by both the ACA and posterior cerebral artery (PCA)(40.9%). Only 3.6% cases were involved only the PCA. The median size of infarcts was 5.0 mm (range, 0.2–20.0).

**Table 1:** Baseline characteristics of the MIS in neurologic outpatients (n = 753).

Characteristic	Value
Age,(years, mean ±SD)	60 ±11.6
Female sex, n(%)	374(49.7)
Nonfocal neurologic symptoms, n(%)	678(90%)
Dizziness/vertigo	201(29.6)
Headache/migraine variation	165(24.3)
Headache with dizziness	181(26.7)
Others	131(19.3)
Focal neurologic symptoms, n(%)	75(10%)
No. of initial NIHSS score met MIS, n(%)	
0 score	602(80.0)
1 score	119(15.8)
2 score	28(3.7)
3 score	4(0.5)
MRI findings, n(%)	
DWI positive lesions	172(43.0)
FLAIR positive lesions	753(100.0)
Median time of onset to MRI (days, range)	15(1898.9)
Median number of lesions (range)	8(1–110)
Median size of infarcts (mm,range)	5.0(0.3–20.0)
Multiple infarction(%)	685(91.6)
Brain atropy(%)	12(1.6)
Leukoaraiosis(%)	49(6.6)
Location of MIS	
ACA	418(55.5)
PCA	27(3.6)
ACA+PCA	308(40.9)

MIS = minor ischemic stroke; DWI = diffusion-weighted imaging; FLAIR = fluid-attenuated inversion recovery; ACA = anterior circulation artery; PCA = posterior circulation artery.

Of the MIS patients, 303(40.2%) were identified to have MetS, while 450 (59.8%) did not. The distribution of individual components MetS criteria in patients with MIS is exhibited in Table 2. In MIS patients with MetS, the most frequent single criterion that was met was a hypertension (91.1%), followed by an increased blood glucose and raised TC.

**Table 2:** Distribution of individual components meeting MetS criteria in MIS patients with or without MetS.

Variable	MIS with MetS (N = 303)	MIS without MetS (N = 450)	*P* Volume
MetS individual components			
Abdominal obesity, n (%)	93(30.7)	81(18.0)	<0.001
Hypertension, n (%)	276(91.1)	367(81.6)	<0.001
Raised fasting plasma glucose	234(77.2)	17(3.8)	<0.001
Raised TG, n (%)	205(67.7)	189(42.0)	<0.001
Decreased HDL-C	37(10.4)	26(4.7)	0.002
No. of MetS components met			
0	0(0)	33(7.3)	
1	0(0)	203(45.1)	
2	0(0)	214(47.6)	
3	235(77.2)	0(0)	
4	56(18.4)	0(0)	
5	12(4.0)	0(0)	

MIS = minor ischemic stroke; MetS = metabolic syndrome; TG = triglycerides; LDL-C = low density lipoprotein cholesterol.

On those tranditional risk factors, MetS was significantly correlated with the abdominal obesity (30.7%v.s 18.0%), hypertension (91.1% v.s 81.6%), diabetes mellitus (18.8% v.s 0.01%), increased blood glucose (6.9±2.4 v.s 5.0±0.4), and dyslipidemia (78.2% v.s 48.2%). The MIS patients with MetS were significantly more likely to present with SVE (50.5% v.s 11.3%) than those without MetS (Table 3).

**Table 3:** The results of univariate analysis in patients with MetS and without MetS.

Variable	MIS with MetS (N = 303)	MIS without MetS (N = 450)	*P* Volume
Male gender, n (%)	165(54.5)	214(47.6)	0.063
Age (years,mean ±SD)	60.6±10.2	59.9±12.0	0.683
BMI(kg/m2, mean ±SD)	24.7±3.0	23.5±3.2	0.004
Abdominal obesity, n (%)	93(30.7)	81(18.0)	<0.001
Hypertension, n (%)	276(91.1)	367(81.6)	<0.001
Diabetes mellitus, n (%)	57(18.8)	6(0.01)	<0.001
Heart disease, n (%)	10(0.03)	7(0.02)	0.114
Atrial fibrillation, n (%)	9(0.03)	6(0.01)	0.115
Smoking, n (%)	106(35.0)	118(26.2)	0.979
Alcohol drinker, n (%)	87(32.0)	125(27.8)	0.780
Blood glucose (mmol/l, mean ±SD)	6.9±2.4	5.0±0.4	<0.001
Dyslipidemia, n (%)	237(78.2)	217(48.2)	<0.001
SBP, mm Hg (mean ±SD)	153.1±22.1	150.5±21.2	0.336
DBP, mm Hg (mean ±SD)	97.4±10.9	94.8±11.6	0.073
Number of lesions on MRI (mean ±SD)	11±12.4	10±14.3	0.828
Size of lesion on MRI (mm, mean ±SD)	1.2±1.7	1.0±1.6	0.549
Initial NIHSS score (mean ±SD)	0.3±0.6	0.2±0.5	0.349
Subsequent vascular event, n (%)	153(50.5)	51(11.3)	<0.001
Mortality, n (%)	3(1.0)	4(0.9)	0.887

MIS = minor ischemic stroke; MetS = metabolic syndrome; BMI = body mass index; SBP = systolic blood pressure; DBP = diastolic blood pressure.

Outcome data were available among patients with MIS. The risk of death was similarly low in patients with or without MetS (1.0% v.s 0.8%) during the follow-up. Three patients with MetS died from the progression of stroke, and two additionally died from stroke itself. Two patients without MetS died from lung cancer.

Adjusted for age and sex, only three individual MetS components were significantly associated with the risk of developing MIS associated SVE (Table 4). These included hypertension (RR,2.6; 95%CI, 1.020–6.544;p = 0.045), high blood glucose (RR, 2.8; 95% CI, 1.938–4.113; p<0.001), and increased GC (RR, 3.2; 95% CI, 1.753–5.603; p<0.001), in addition to older age (RR, 1.0; 95% CI, 1.022–1.072; p<0.001). The frequency of the SVE in MIS patients increased over the number of positive individual MetS factors (from 42.9% at a threshold of three MetS criteria to 91.7% at a threshold of five MetS criteria) (Figure A in S1 File).

**Table 4:** MetS individual components and the risk of SVE in patients with MIS during follow-up (n = 753), adjusted for age and sex.

Variable	MIS with SVE (N = 204)	MIS without SVE (N = 549)	RR (95% CI)	*P* Volume
Male gender, n (%)	105(51.5)	270(49.2)	1.3(0.797–2.189)	0.280
Age (y, mean ±SD)	63.4±11.0	59.0±11.2	1.0(1.022–1.072)	0.000
MetS, n (%)	156(76.5)	147(26.8)	1.6(0.818–3.196)	0.176
Abdominal obesity, n (%)	63(30.9)	111(20.2)	1.1(0.968–1.147)	0.255
Hypertension, n (%)	189(92.6)	453(82.5)	2.6(1.020–6.544)	0.045
Blood glucose, (mmol/l, mean ±SD)	7.2±2.9	5.2 ±0.7	2.8(1.938–4.113)	<0.001
TG, n (%)	158(77.3)	273(49.7)	3.2(1.753–5.603)	<0.001
HDL-C	31(15.4)	21(3.8)	1.0(0.411–2.643)	0.930

MIS = minor ischemic stroke;SVE = subsequent vascular event; MetS = metabolic syndrome; TG = triglycerides; HDL-C, = High density lipoprotein cholesterol; RR, = risk ratio; CI = confidence interval.

**Fig 2:**
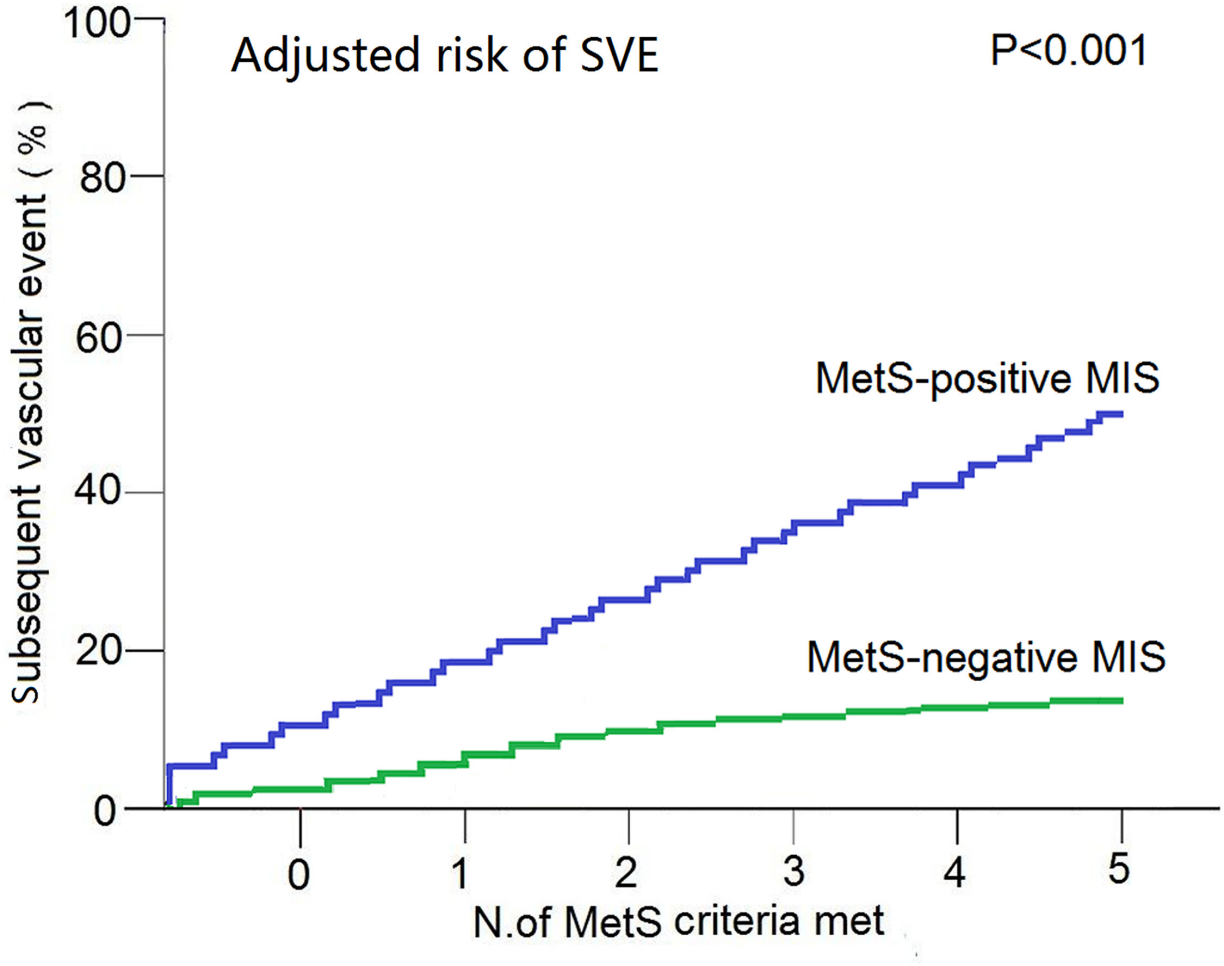
Adjusted risk of SVE among Patients with MIS, According to Number of MetS Criteria Met. Adjusted risk of SVE show that MIS patients with MetS had significantly frequent SVE than those MIS patients without MetS during the 6-months follow-up (Risk ratio = 9; 95% CI, 20.29–39.31; P = 0.000).

In correlate analysis, SVE in MIS patients were positively correlated with the frequency of individual MetS factors (r = 0.466, P<0.001). Using a logistic model adjusted to include the number of MetS criteria from 0 to 5, the risk radio for SVE was also significantly associated with three additional MetS criterion (50.5% verse 11.3%; RR,9.0; 95%CI, 5.677–14.46; P<0.001) (Fig 2).

Cox proportional risk analysis demonstrated that only the MetS (RR, 3.3; 95% CI, 1.799–6.210; p<0.0001), older age (RR, 1.0; 95% CI,1.001–1.048; p = 0.039),and high blood glucose(RR,1.1;95%CI,1.007–1.187;p = 0.033) were significantly related to SVE in patient with MIS(Table 5).

**Table 5:** Cox analysis of the association of outcome for MIS patients with or without SVE.

Variable	MIS with SVE (N = 204)	MIS without SVE (N = 549)	RR (95%CI)	*P* Volume
Age (years,mean ±SD)	63.4±11.0	59.0±11.2	1.0(1.001–1.048)	0.039
Blood glucose, (mmol/l, mean ±SD)	7.2±2.9	5.2 ±0.7	1.1(1.007–1.187	0.033
MetS, n (%)	156(76.5)	147(26.8)	3.3(1.799–6.210)	<0.001

MIS = Minor ischemic stroke; SVE = subsequent vascular event; MetS = metabolic syndrome;RR = risk ratio; CI = confidence interval.

## Discussion

This study assessed 1361 neurology outpatients. 753(55.3%) patients with recent MIS were identified using MRI, and 80% of these patients had a score of 0 using NIHSS criteria. 40.2% of MIS patients fulfilled three or more MetS criteria at their first-ever visit, supporting the conclusion that the MetS factors assessed in this study may be highly prevalence in MIS patients.

Previous hospital-based studies have suggested that MetS is independently associated with ischemic stroke [10], [11],[13]. Moreover, some previous hospital-based studies showed that MetS factors were significantly higher in patients with serious ischemic stroke compare to controls [13], [14], and patients with these factors were at higher risk of recurrent stroke than those without MetS [15],[21]. Our study confirmed that neurologic outpatients with recently diagnosed MIS may also be at risk of Mets. Thus, we believe that MetS is a contributing factor in conditions ranging from severe stroke to MIS.

Previous studies demonstrated that the relationship between MetS factors and ischemic stroke were independent. Nira *et al* indicated that individuals with MetS without diabetes exhibited a 1.49-fold increased risk of ischemic stroke or TIA [22]. More recently, one study showed that only the hypertensive trait among MetS factors was associated with a significantly elevated risks of ischemic stroke [23]. Our data demonstrated that MIS patients with MetS had higher abdominal obesity, a hypertension, a higher diabetes mellitus ratio or increased blood glucose, higher dyslipidemia ratio, and more frequent SVE than those without MetS. These finding were similarly recognized in previous community studies [3], [4]and in a recent study[24]. However, using a multivariable analysis, the older age, hypertension, increased blood glucose, and higher triglycerides were found to be independent predictors of SVE. A recent study showed that serum hyperglycemia contributed to poor functional outcomes [25]. Furthermore, some studies have shown that older patients with ischemic stroke are more likely to have worse outcome [26], [27]. In our current study, a Cox proportional risk analysis confirmed that MetS, older age, and high blood glucose were significantly related to SVE in patient with MIS. Moreover, MetS was identified to be the biggest risk factor for development of SVE in MIS patients (RR, 3.3). On adjusted analysis, the risk of SVE was also significantly associated with three additional MetS criterion. Our current findings support the use of three criteria as a cutoff point for the identification of at-risk patients. From a practical perspective, prophylactic measures for outpatients diagnosed with MetS with MIS are currently less widely implemented than for hospitalized patients with serious ischemic stroke. Therefore, we believe that these findings are likely to be essential for planning and implementation of prevention and treatment programs.

Limitations of this retrospective cohort study should be mentioned. Firstly, some previous studies showed that a higher proportion of females had MetS compared to males [28], [29]. However, our current study did not result in a difference between sexes. As our study was based on a randomly selected subgroup of Neurologic outpatients, the presence confounding could not entirely be excluded. However, of the included study population, 94% of subjects lived in one of 38 villages or towns, while only 6% were from the county town. This allowed us to determine the proportion of patients with MIS in the community who were admitted to the neurologic outpatient clinic, suggesting at most a very small bias in our study population. Secondly, the frequency of SVE has previously been correlated with the presence of intracranial artery stenosis or carotid plaque [30], [31], suggesting that these factors may contributed to SVE in MetS patients with uncertain diagnoses. Furthermore, the presence of asymptomatic intracranial artery stenosis due to MetS has been confirmed by recent study [32]. Unfortunately, most of the MIS patients with MetS in the present study did not have available vascular imaging and transcranial color-coded Doppler sonography data. In addition, the diagnosis of MetS was based on a single assessment comprising a number of MetS factors. Some vascular risk factors assessed at the 6 months follow-up may have been affected by medication provided over this period. This may have reduced the associations found in this study. However, the individual MetS factors pertaining to bodily measurements or laboratory data were prospectively collected and were less likely to be biased. Therefore, we believe that the high prevalence of MetS in MIS patients was unlikely to be overestimated in our study.

In conclusion, our outpatient-based study revealed that the prevalence of MetS was high in patients with MIS, and that these patients were more likely to be at risk of SVE. Further assessing methods to prevent and improve the features of MetS may be important to reduce MIS and associated SVE.

## Supporting Information

S1 File Figure A. The frequency of SVE in MIS patients increased with the number of positive individual MetS factors (from 42.9% at a threshold of three MetS criteria to 91.7% at a threshold of five MetS criteria). Table A. Minimal data set. Multivariate and Cox analysis of the association of outcome for MIS patients with or without SVE.
(DOC)
