# Supplementary material for: Metabolic Syndrome Is a Strong Risk Factor for Minor Ischemic Stroke and Subsequent Vascular Events
Source: PLoS One. 2016 Aug 18;11(8):e0156243. doi: 10.1371/journal.pone.0156243 (PMC4990187; doi:10.1371/journal.pone.0156243)
Supplement: S1 File — Figure A. The frequency of SVE in MIS patients increased with the number of positive individual MetS factors (from 42.9% at a threshold of three MetS criteria to 91.7% at a threshold of five MetS criteria). Table A. Minimal data set. Multivariate and Cox analysis of the association of outcome for MIS patients with or without SVE. (DOC) [file pone.0156243.s001.doc]

## Supporting Information


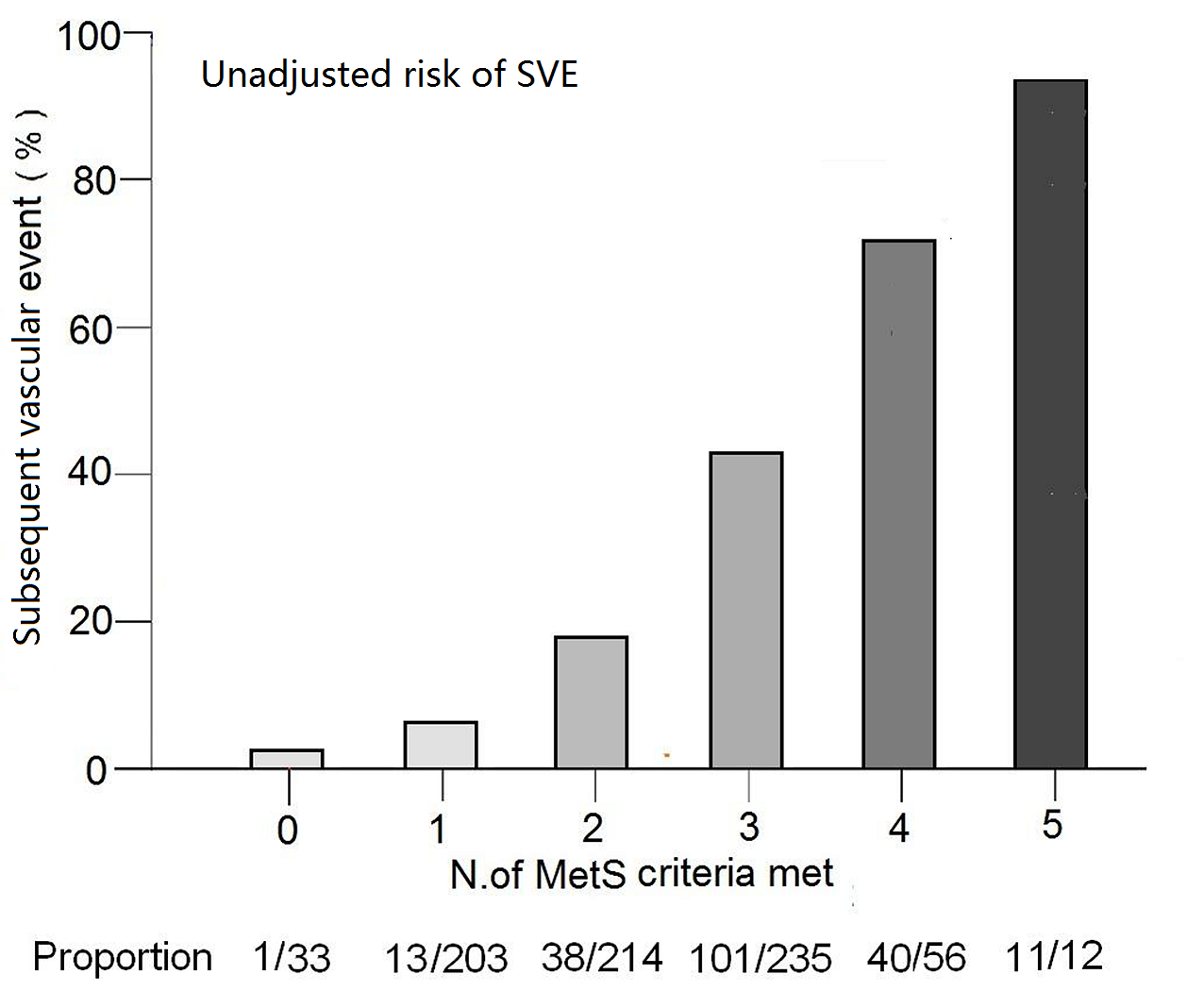
 Figure A.The frequency of SVE in MIS patients increased with the number of positive individual MetS factors (from 42.9% at a threshold of three MetS criteria to 91.7% at a threshold of five MetS criteria)

Table A. Minimal data set. Multivariate and Cox analysis of the association of outcome for MIS patients with or without SVE

| Variable | MIS with SVE  (N=204) | MIS without SVE  (N=549) | RR for Multivariate P  (95%CI) Volume | RR for Cox P  (95%CI) Volume |
| --- | --- | --- | --- | --- |
| Male gender, n (%)  Age (y, mean ±SD)  MetS, n (%)  Abdominal obesity, n (%)  Hypertension, n (%)  BG, (mmol/l, mean ±SD)  TG, n (%)  HDL-C | 105(51.5)  63.4±11.0  156(76.5)  63(30.9)  189(92.6)  7.2±2.9  158(77.3)  31(15.4) | 270(49.2)  59.0±11.2  147(26.8)  111(20.2)  453(82.5)  5.2 ±0.7  273(49.7)  21(3.8) | 1.3(0.8-2.2) 0.280  1.0(1.0-1.1) <0.001  1.6(0.8-3.2) 0.176  1.1(0.9-1.1) 0.255  2.6(1.0-6.5) 0.045  2.8(1.9-4.1) <0.001  3.2(1.7-5.6) <0.001  1.0(0.4-2.6) 0.930 | 1.0(1.0-1.0) 0.039  3.3(1.8-6.2) <0.001  1.1(1.0-1.2) 0.033 |

MIS=minor ischemic stroke;SVE=subsequent vascular event; MetS=metabolic syndrome; BG=Blood glucose; TG= triglycerides; HDL-C,= High density lipoprotein cholesterol; RR,=risk ratio; CI*=*confidence interval
